# Comparative evaluation of pregnancy outcome in gonadotrophin-clomiphene combination vs clomiphene alone in polycystic ovarian syndrome and unexplained infertility–A prospective clinical trial

**DOI:** 10.4103/0974-1208.69341

**Published:** 2010

**Authors:** Shiuli Mukherjee, Sunita Sharma, B N Chakravarty

**Affiliations:** Department of Reproductive Medicine, Institute of Reproductive Medicine, Salt Lake City, Kolkata - 700 106, India

**Keywords:** Clomiphene citrate, fecundity, miscarriage, ovulation induction, uFSH

## Abstract

**OBJECTIVES:**

A large prospective clinical trial was conducted to compare the efficacy of single dose uFSH and clomiphene citrate combination with clomiphene citrate alone for ovulation induction to improve the pregnancy rate.

**MATERIALS AND METHODS:**

The study was a randomized, prospective clinical trial. Totally, 1527 infertile women (4381 cycles) with polycystic ovarian syndrome (*P*COS) (n=911/2573 cycles) and unexplained infertility (n=616/1808 cycles) were randomized into two groups. Group A received single dose of uFSH on D_3_ of menstrual cycle along with clomiphene. Group B received clomiphene only for ovulation induction. We compared the pregnancy rate and miscarriage rate between two groups.

**RESULTS:**

Group A had a pregnancy rate of 17% compared to 8.3% of Group B which was significantly higher (*P*=0.0001). The miscarriage rate was 11% in Group A and 10% in Group B which was not significant (*P*=0.99). Pregnancy rates in PCOS women were 22% in Group A and 9.3% in Group B which shows significantly higher pregnancy rate (*P*=0.0001) in anovulatory infertility. But in unexplained infertility, there was no significant difference in pregnancy rate between Group A (11%) and Group B(6.3%). Miscarriage rates were 8.8% and 9.5% in Group A and Group B, respectively, in PCOS women and 14% and 13% in women with unexplained infertility.

**CONCLUSION:**

Addition of single dose of uFSH improves pregnancy outcome particularly in anovulatory infertility (WHO II). Correction of unexplained infertility may need more than simple correction of possible subtle ovulatory effect.

## INTRODUCTION

For the last couple of decades, clomiphene citrate remained the most popular drug for ovulation induction at the initial stage of management of infertile couples with anovulation (WHO group II) and unexplained infertility.[1,2] Though induction of ovulation was achieved in 42-80% cases depending on the diagnosis,[3] the conception rate averaged 9-13% per cycle through 3-6 cycle.[4,5] The gap between ovulatory and pregnancy rates had variously been attributed to its anti-estrogenic effects on endometrium,[6] cervical mucous,[7] and high LH,[8] resulting in luteal phase dysfunction. Several modifications have been tried to overcome the adverse effects of clomiphene by clomiphene plus therapy, such as clomiphene plus dydrogesterone[9] or clomiphene plus vaginal estradiol and micronized progesterone,[10] clomiphene plus dexamethasone,[11] clomiphene plus human menopausal gonadotrophin,[12] clomiphene plus HCG plus IUI,[13] clomiphene plus metformin[14] etc. Though the maximum cumulative pregnancy rate was achieved around 31% through 3-6 consecutive cycles,[15] the fecundity rate remained 8-14%.[16] Thus, our study was designed to improve the fecundity rate by adding single dose gonadotrophin with usual five days clomiphene stimulation along with luteal phase progesterone support. The intention was to develop a CC plus regimen convenient for general gynecologists to get an improved pregnancy outcome at a lower cost and simultaneously avoiding the risk of ovarian hyper stimulation syndrome commonly associated with continuous gonadotrophin therapy.

## MATERIALS AND METHODS

### Subject selection

The randomized prospective controlled trial was conducted from February 2008 to January 2009 at the Institute of Reproductive Medicine (IRM), Kolkata, India. Approval was obtained from the institutional research ethics board and written consent was taken. A total of 1527 women had 4381 cycles initiated. There were 911 women with PCOS who started 2573 treatment cycles and 616 women with unexplained infertility who had 1808 cycles initiated.

In women with PCOS who did not have spontaneous menstrual cycle, withdrawal bleeding was induced by administering oral medroxyprogesterone acetate 10 mg twice daily for 5 days. PCOS was diagnosed by Rotterdam criteria.[18] All these women had both ultrasound and endocrine assessment done prior to the beginning of treatment. Those patients who had previously failed to conceive or ovulate on clomiphene up to a dose of 150 mg daily for five days were excluded from the study. Patients with endometriosis and previous history of ovarian drilling were also excluded.

Unexplained infertility was diagnosed in couples when the semen analysis was normal [volume 2–5 ml, concentration >20 million/ml, >50% total motility, >30% normal forms (WHO 1999)].[19] These patients had at least one tube patent and there were no significant intrauterine or pelvic abnormalities demonstrated on laparoscopy and hysteroscopy.

All couples with PCOS also had two semen analysis to exclude male factor infertility. The female partner had a laparoscopy done to exclude tubal or pelvic factor infertility and endometriosis. Specific inclusion criteria were normal TSH and Prolactin levels for all patients.

Patients were randomized into two groups by sealed envelope, viz. Group A including those who received single dose of uFSH on day 3 along with CC and Group B consisting of those who received only CC. The study group or Group A included 460 women with PCOS who had 1260 cycles initiated and 379 women with unexplained infertility who had 1107 cycles initiated. The control group or Group B included 451 women with PCOS who had 1313 cycles initiated and 237 women with unexplained infertility who had 701 cycles initiated.

### Ovulation induction protocol

Patients in Group A received CC (Ovofar, Organon, Mumbai, India) 100 mg daily from day 3 to 7 of menstrual cycle along with one ampoule of uFSH 75 IU (Follimon, LG Life Sciences India Pvt. Ltd.) on day 3 of each cycle intramuscularly. Women in Group B received 100 mg of CC only from day 3 to 7 of menstrual cycle. All patients were asked to do timed intercourse (TI) in between day 10 to 20. Further all of them were treated with either micronized progesterone (Orgagest, Organon, Mumbai, India) 400 mg intravaginally or dydrogesterone 20 mg orally (Duphaston, Solvay Pharma India Limited, India) daily from day 16 to day 25 of same menstrual cycle for luteal phase support. Treatment continued for maximum of three cycles for pregnancy to occur. A urine pregnancy test was performed if the expected menstrual cycle were delayed. Pregnancy was defined as a rising concentration of serum βHCG and a gestational sac with fetal pole and heart beat on ultrasound at 6 weeks of gestation. An ongoing pregnancy was defined as a pregnancy after 20 weeks of gestation. The primary outcome measures pregnancy rate and miscarriage rate.

### Hormone assay

Serum FSH, LH, TSH and prolactin concentration were measured by two side chemiluminescent sandwich immunoassay system. All samples were assayed in duplicate. LH and FSH were expressed in terms of the reference standards (WHO 2^nd^ IS 94/632 and WHO 2^nd^ IS 80/552, respectively). Assay sensitivity for FSH was 0.3mIU/ml and for LH was 0.07mIU/ml.

### Statistics

The effectiveness of a single FSH dose plus CC compared with CC alone was expressed as a relative ate ratio (RR) for pregnancy and miscarriage rate. Further statistical analysis was carried out using the Fisher Exact test while comparing the outcomes. Student’s t-test was used to assess different cycle parameters between the two groups. Data were expressed as mean±SD. The statistical package SPSS 10 was used. Significance of the test was performed at the 5% level (*P*<0.05).

## RESULTS

### Patient characteristics

The clinical profiles including age, duration of infertility, BMI, baseline FSH, LH and TSH, Prolactin levels among the 1527 women are summarized in Table 1. The mean characteristics were almost comparable in two groups. There was no significant difference between the variables in two groups. Variables were equally distributed in Group A and Group B.

**Table 1:** Base line characteristics of infertile women randomly subjected to two different groups for ovulation induction protocol: Group A received CC+ single dose uFSH, group B received CC alone (mean±SD)

Clinical characteristics	Group A CC+ uFSH protocol (n=839)	Group B CC protocol (n=688)
Mean age (years)	30±4.8	30±4.5
Duration of marriage (years)	6.3±4.1	6.1±3.5
Mean BMI (kg/m^2^)	24±3.7	23±3.7
TSH (mIU/ml)	3.2±1.0	3.2±1.0
FSH (mIU/ml)	5.6±1.9	5.7±1.8
LH (mIU/ml)	6.8±3.4	6.3±2.7
PRLmIU/ml	18±3.8	18±3.7

### Cycle characteristics

There were 2573 treatment cycles completed in women with PCOS and 1808 cycles in women with unexplained infertility. Table 2 shows the number of women and number of treatment cycles in each of study and control groups. A total of 50 women among the 460 women with PCOS who had undergone a stimulation of uFSH + CC conceived after first cycle. Another 20 women conceived after two cycles and 32 took three cycles to conceive. Remaining 358 women completed three cycles but did not conceive. In unexplained infertility, 9 conceived after first cycle but 12 women needed two cycles and 20 women required three cycles to conceive. Remaining 338 women had three cycles completed who remained non-pregnant in Group A or Study group. In Group B or Control group (women getting CC only stimulation) 16 women conceived after one cycle, 8 women after two cycles and 18 after three cycles. Remaining 409 completed three cycles and were non-pregnant. Table 3 shows the number of cycles per women had in both the groups.

**Table 2:** Number of patients and number of treatment cycles in both groups

	Group A uFSH+CC			Group B CC			
	All	PCOS	UI	All	PCOS	UI
No. of patients	839	460	379	688	451	237
No. of treatment cycles	2367	1260	1107	2014	1313	701

PCOS= Poly cystic ovarian syndrome, UI=Unexplained infertility

Table 4 represents the outcome measured between two groups. The pregnancy rate in Group A (17%) was significantly higher than in Group B (8.3%) *P*=0.0001 [RR 2.1; 95% CI 1.5 to 2.7]. The spontaneous miscarriage rates were comparable, 12% and 11% respectively (*P*=0.99) not significant.

**Table 3:** Number of cycles per patients in both groups

No. of cycles	Group A uFSH +CC				Group B CC			
	PCOS (n=460)		UI (n=379)		PCOS (n=451)		UI (n=237)	
	Pregnant (n=102)	Non-pregnant (n=358)	Pregnant (n=41)	Non-pregnant (n=338)	Pregnant (n=42)	Non-pregnant (n=409)	Pregnant (n=15)	Non-pregnant (n=222)
1	50	-	9	-	16	-	2	-
2	20	-	12	-	8	-	6	-
3	32	358	20	338	18	409	7	222

**Table 4:** Comparison of pregnancy and miscarriage rate between Group A and Group B

Cycle parameters	Group A CC+uFSH protocol (n=839)	Group B CC-protocol (n=688)	Relative rate ratio (95% CI)	*P* value
Pregnancy rate	17% (143)	8.3% (57)	2.12 (1.53, 2.74)	0.0001
Miscarriage rate	12% (17)	11% (6)	1.12 (0.46, 2.71)	0.99

The two tailed *P* value was calculated by Fisher Exact Test. CI=confidence interval

Data were sub categorized according to cause of infertility. The pregnancy outcomes for the 911 PCOS women in Group A and Group B are summarized in Table 5. In women with PCOS, the pregnancy rate in Group A (22%) was significantly higher than in Group B (9.3%) *P*=0.0001 [RR 2.4; 95% CI 1.7 to 3.3]. The spontaneous miscarriage rates were comparable at 8.8% and 9.5% respectively (*P*= 0.99).

**Table 5:** Comparison of cycle parameters in PCOS patients

Cycle parameters	Group A CC+uFSH protocol (n=460)	Group B CC-protocol (n=451)	Relative rate ratio (95% CI)	*P* value
Pregnancy rate	22% (102)	9.3% (42)	2.38 (1.70, 3.32)	0.0001
Miscarriage rate	8.8% (9)	9.5% (4)	0.92 (0.30, 2.84)	0.99

The two tailed *P* value was calculated by Fisher Exact Test. CI=confidence interval

For the 616 women with unexplained infertility, the pregnancy outcomes in Group A and Group B are summarized in Table 6. In women with unexplained infertility, there was no evidence for a quite statistically significant difference in pregnancy rate; the pregnancy rate in Group A was 11% and in Group B 6.3% *P*=0.07 [RR 1.7; 95% CI 0.97 to 3.0]. The spontaneous miscarriage rates were also comparable, 14% and 13% respectively (*P*=0.99).

**Table 6:** Comparison of cycle parameters in patients with unexplained infertility

Cycle parameters	Group A CC+uFSH protocol (n=379)	Group B CC-protocol (n=237)	Relative rate ratio (95% CI)	*P* value
Pregnancy rate	11% (41)	6.3% (15)	1.70 (0.96, 3.01)	0.07
Miscarriage rate	14% (6)	13% (2)	1.09 (0.24, 4.85)	0.99

The two tailed *P* value was calculated by Fisher Exact Test. CI=confidence interval

## DISCUSSION

The pregnancy rate in Group A was found to be significantly higher as compared to Group B (*P*=0.0001) in this study. This is in good agreement with few other studies which have compared CC+HMG with CC.[20–27] The clinical pregnancy rates were 13-23%,[20–22] which corroborates to our study where we have got 17% overall pregnancy rate and 22% among PCOS women on adding one dose of uFSH on day 3. Several prospective randomized trials have used sequential gonadotrophin either HMG or FSH from day 7 through 9 along with CC with the idea of rescuing the non-dominant follicles that normally undergo atresia due to suppression of FSH secretion by rising estrogen concentration produced by the dominant follicle.[20–27] Some of those studies have claimed that the pregnancy rates with sequential clomiphene—HMG were almost equal to HMG alone.[20,28] Thus, such regimen has the advantage of controlled ovarian hyperstimulation (COH) at a lower cost and less cycle monitoring.[28]

Initially the sequential use of hMG following CC was postulated by Kistner *et al*[23] in 1966 to improve the ovulation as well as the pregnancy rate. The explanation for increased fecundity when HMG is administered after clomiphene, compared to clomiphene alone, was related to both increased numbers of pre-ovulatory follicles and a doubling of the implantation rate per follicle.[20,24] The oestradiol level per follicle also increased to nearly double for clomiphene-HMG group compared to clomiphene alone.[26,27] It is established that the level of oestradiol is related to fecundity. Higher oestradiol level may improve the quality of either the oocyte or cumulus mass within the follicle, or of the tubal milieu for fertilization and preimplantation embryonic development or of the endometrium. In this study, our main target was to recruit the co-dominant follicles earlier at follicular phase and therefore enhancing the chances of pregnancy. And surely we have gone a step ahead of others by decreasing the duration and dose of gonadotrophin required for ovulation induction, yet maintaining expected pregnancy rate by single dose uFSH on day 3. Most of the previous studies have used 3 or 4 doses of HMG/FSH with clomiphene.[25–27] None of the studies have used single dose gonadotrophin on day 3.

Thus, in this study, we have achieved a pregnancy rate equal to them at a more cost effective single dose regimen.

Among the sub group of PCOS women, we have shown a pregnancy rate of 22% in the CC+uFSH combined arm compared to that of 9.3% in CC only arm. Thus, in PCOS women, the pregnancy rates have significantly increased in Group A compared to Group B (*P*=0.0001). This is well corroborated with few other studies who achieved a pregnancy rate of 14-20% on PCOS patients following a protocol of adding FSH 75IU on day 3 and day 8 or continuously from day 7 till the day of hCG.[28,29]

It is well established that CC indirectly stimulates GnRH secretion which increases the release of both FSH and LH. This increase in LH, whose basal level is often already high in women with PCOS, may compromise pregnancy rates in those receiving CC.[8] Addition of uFSH with CC can negate the detrimental effect of LH on pregnancy and miscarriage, and therefore increases the pregnancy rate in PCOS women. Our observation has made the both ends meet by increasing pregnancy rate to 22% and decreasing the miscarriage rate from 9.5% (Group B) to 8.8% (Group A) though not significant.

The treatment of couples with unexplained infertility was less successful using this treatment. Though the pregnancy rate in Group A (11%) was well above that of Group B (6.3%), the difference was not significant (*P*=0.07) or it shortly misses the level of significance. This result compares favorably with few other reported series.[30,31]

We also found no significant difference in the miscarriage rate between two groups in women with unexplained infertility (*P*=0.99). These indicate that women with unexplained infertility do have some uncharacterized defects which is only partially corrected with ovarian stimulation.[31]

## CONCLUSION

After a long gap on comparative studies in the area of CCHMG/FSH versus CC alone, this study could have brought some fresh air and ray of hope on using combination protocol for ovulation induction in a larger scale. Addition of a single dose of uFSH does have a potential supportive effect on pregnancy rate compared to that of CC alone. Thus, the possibility of replacing CC as first-line treatment particularly for anovulatory infertility with CC+uFSH therapy needs to be substantiated. We need more RCTs throughout the countries to come to a conclusion on achieving the best outcome on infertility cases.
